# Molecular Characterization and Immune Protection of a New Conserved Hypothetical Protein of *Eimeria tenella*

**DOI:** 10.1371/journal.pone.0157678

**Published:** 2016-06-16

**Authors:** Qi Zhai, Bing Huang, Hui Dong, Qiping Zhao, Shunhai Zhu, Siting Liang, Sha Li, Sihan Yang, Hongyu Han

**Affiliations:** 1 Shanghai Veterinary Research Institute, Chinese Academy of Agricultural Sciences, Key Laboratory of Animal Parasitology of Ministry of Agriculture, Minhang, Shanghai, PR China; 2 Jiangsu Co-innovation Center for Prevention and Control of Important Animal Infectious Diseases and Zoonoses, Yangzhou, PR China; Instituto Butantan, BRAZIL

## Abstract

The genome sequences of *Eimeria tenella* have been sequenced, but >70% of these genes are currently categorized as having an unknown function or annotated as conserved hypothetical proteins, and few of them have been studied. In the present study, a conserved hypothetical protein gene of *E*. *tenella*, designated *Et*CHP559, was cloned using rapid amplification of cDNA 5'-ends (5'RACE) based on the expressed sequence tag (EST). The 1746-bp full-length cDNA of *Et*CHP559 contained a 1224-bp open reading frame (ORF) that encoded a 407-amino acid polypeptide with the predicted molecular weight of 46.04 kDa. Real-time quantitative PCR analysis revealed that *Et*CHP559 was expressed at higher levels in sporozoites than in the other developmental stages (unsporulated oocysts, sporulated oocysts and second generation merozoites). The ORF was inserted into pCold-TF to produce recombinant *Et*CHP559. Using western blotting, the recombinant protein was successfully recognized by rabbit serum against *E*. *tenella* sporozoites. Immunolocalization by using *Et*CHP559 antibody showed that *Et*CHP559 was mainly distributed on the parasite surface in free sporozoites and became concentrated in the anterior region after sporozoites were incubated in complete medium. The *Et*CHP559 became uniformly dispersed in immature and mature schizonts. Inhibition of *Et*CHP559 function using anti-r*Et*CHP559 polyclonal antibody reduced the ability of *E*. *tenella* sporozoites to invade host cells by >70%. Animal challenge experiments demonstrated that the recombinant *Et*CHP559 significantly increased the average body weight gain, reduced the oocyst outputs, alleviated cecal lesions of the infected chickens, and resulted in anticoccidial index >160 against *E*. *tenella*. These results suggest that *Et*CHP559 plays an important role in sporozoite invasion and could be an effective candidate for the development of a new vaccine against *E*. *tenella*.

## Introduction

Chicken coccidiosis is a protozoal disease caused by infection with several *Eimeria* species and leads to high annual economical losses in the poultry industry due to high morbidity, reduction of body weight, and treatment costs [1]. At present, the control of *Eimeria* infection is still based mainly on anticoccidial drugs and live vaccines. However, these measures have been restricted by some reasons, for example, the consumer attention to food safety, the rise of drug resistance, and the high production expenses etc. Therefore, new cost-effective anticoccidial control strategies need to be developed [2,3].

*Eimeria* are classified in the phylum Apicomplexa, which contains obligate intracellular parasites including medical and veterinary pathogens such as *Plasmodium*, *Toxoplasma*, *Cryptosporidium*, *Neospora* and *Sarcocystis*. These protozoan parasites are characterized by a peculiar organelle complex located at the apical end [4]. The life cycle of *Eimeria* is complex and comprises three distinct phases: sporogony, schizogony and gametogony [5]. These phases involve different life cycle forms which have different morphological characteristics and habitats, including unsporulated oocysts, sporulated oocysts, sporozoites, trophozoites, merozoites and gametocytes. The levels of gene expression among these stages often differ greatly and this increased the difficulty of developing the cost-effective subunit vaccines.

The genomes of all seven *Eimeria* species that infect chickens have been sequenced and each *Eimeria* species expresses between 6000 and 9000 proteins throughout its life cycle [6]. However, the annotation of coding sequences is still a major challenge. Among seven *Eimeria* species, *Eimeria tenella* is one of the most important species, causing cecal hemorrhage and high mortality. A lot of research on *E*. *tenella* has been reported. But more than 70% of the *E*. *tenella* genes are currently categorized as unknown function or annotated as conserved hypothetical proteins [5], so some conserved proteins maybe important for invasion, development and the life cycle of *E*. *tenella*. Until now, few genes of the conserved hypothetical proteins have been studied and tested for their function and immunogenicity, although there are nucleotide sequences in GenBank.

In our previous research, some expressed sequence tags (ESTs) that encoded potential interaction proteins with *Et*AMA1were obtained by screening from the yeast two-hybrid cDNA library of *E*. *tenella* sporozoites using *Et*AMA1 as bait (data unpublished). BLAST searches showed that a protein encoded by ESTs559 (GeneBank accession NO.JZ905777) was annotated as a conserved hypothetical protein. In this study, we used the rapid amplification of cDNA ends (RACE) technique to clone the full-length cDNA sequence based on the EST sequence described above. Then, we identified the molecular characterization, and the immunogenicity of the recombinant protein was checked through chicken challenge experiments.

## Materials and Methods

### Ethics statement

The protocol was approved and authorized by the Animal Care and Use committee of the Shanghai Veterinary Research Institute, Chinese Academy of Agricultural Sciences.

### Animals

Newly hatched 1-day-old Chinese Pudong yellow chickens purchased from the hatchery of Min You (Shanghai, China) were raised under coccidian-free conditions. The New Zealand white rabbits were purchased from the Shanghai Experimental Animal Center, Chinese Academy of Sciences. We examined the coccidia from rabbits feces for three days and judged the rabbits as coccidian-free. Then the rabbits were also raised under coccidian-free conditions.

### Parasite propagation and purification

The Shanghai strain of *E*. *tenella* was isolated from a sample collected on a chicken farm in Shanghai, China in the 1980s and subsequently maintained in our laboratory [7]. Parasites were propagated by passage through coccidia-free 2-week-old chickens as previously described [8]. Unsporulated oocysts (UO) were obtained after infected chickens with 5× 10^4^ sporulated oocysts per bird and undergone sporulation to become sporulated oocysts (SO). Then unsporulated oocysts and sporulated oocysts were purified using standard procedures [9,10]. Sporozoites (Spz) were purified from cleaned sporulated oocysts by *in vitro* excystation [9]. Second-generation merozoites(Mrz) were collected from the cecal mucosa of chickens at 115 h post inoculation (p.i.) with 10^5^ sporulated oocysts per bird. Briefly, the ceca contents were discarded. Then the ceca were rinsed with PBS and cut into small pieces. After enzymatic digestion, the merozoites were released from the ceca and purified by filtration, centrifugation, erythrocyte disruption and Percoll density gradient centrifugation [11].

### Total RNA extraction and cDNA synthesis

Total RNA of *E*. *tenella* sporozoites was extracted by using TRIzol (Takara, Dalian, China). To avoid DNA contamination, the extracted RNA preparations were additionally treated with RNase-free DNase Ι (Takara) for 30 min at 37°C according to the manufacturer instructions and then inactivated by heating at 75°C for 10 min. RNA was quantified by NanoDrop 2000C (Thermo Scientific, Waltham, MA, USA) and its integrity was verified by 1% agarose denaturing formaldehyde-Dured gel electrophoresis. cDNA was synthesized from the total RNA using an M-MLV Reverse Transcriptase kit (Invitrogen, Beijing, China) with Oligo dT primers. The cDNA was then used as a template for further study.

### Molecular cloning of the *Et*CHP559 full-length cDNA

A yeast two-hybrid cDNA library from sporozoites of *E*. *tenella*, constructed in our laboratory previously, was screened with the bait *Et*AMA1 protein. One of the putative positive clones (Clone No.Et559, GenBank accession No.JZ905777) expected to be the interaction protein with *Et*AMA1 was chosen for further analysis. The 701 bp EST sequence of the clone had 100% identity with *E*. *tenella* hypothetical protein (XM_013376471.1) in NCBI. It contains a poly(A) in the 3' end, so the full-length 5'-ends of the cDNA for the gene were obtained by 5'RACE using GeneRacer kits (Invitrogen). GR5P and GR5N primers supplied with the kit and the GS5P and GS5N gene-specific primers listed in Table 1 were used to amplify the 5' flanking sequence. And gene-specific primers were designed on the basis of the EST sequence. Amplified fragments were gel purified (Qiagen, GmbH, Hilden, Germany) and cloned into the pGEM-T-easy vector (Promega, Madison, WI, USA), and sequenced. After aligning and assembling the resulting sequences with the original EST sequence, the full-length cDNA sequence of the CHP559 gene was obtained and submitted to NCBI GenBank (accession number: KT318394).

**Table 1:** Primer sequences used in this study.

Primer ID	Primer sequences
GR5P (GeneRace 5' Primer)	5'-CGACTGGAGCACGAGGACACTGA-3'
GR5N (GeneRace 5' Nested Primer)	5'-GGACACTGACATGGACTGAAGGAGTA-3'
GS5P (Gene-specific 5' Primer)	5'-ACCTTCGACCGCTGCCGCTCGTGTTCCT-3'
GS5N (Gene-specific 5' Nested Primer)	5'-CCGCTGCCGCTCGTGTTCCTTTTCCCAT-3'
RTS (qPCR EtCHP559 Sense primer)	5'-AGTTCCCTCCGAAGTCTATCTCCTC-3'
RTA (qPCR EtCHP559 Antisense primer)	5'- GAAGAACCCCCACTGGAGCCGCAAC-3'
18S (qPCR 18s rRNA Sense primer)	5'-TGTAGTGGAGTCTTGGTGATTC-3'
18A (qPCR 18s rRNA Antisense primer)	5'-CCTGCTGCCTTCCTTAGATG-3'
β-actinS (qPCR β-actin Sense primer)	5′-GGATTGCTATGTCGGCGATGA-3′
β-actinA (qPCR β-actin Antisense primer)	5′-ACACGCAACTCGTTGTAGAAAGTG-3′
GAPDHS (qPCR GAPDH Sense primer)	5′-CGCCACCTAAGGACGATA-3′
GAPDHA (qPCR GAPDH Antisense primer)	5′-TGCCAAGGGAGCCAAGCA-3′
β-tublinS (qPCR β-tublin Sense primer)	5′- GGGGGTCTGCGACATCCCGCCAAAG- 3'
β-tublinA (qPCR β-tublin Antisense primer)	5′–AACTCCATTTCGTCCATACCCTCTC- 3'
HD1(Forward primer)	5'-GCGGATCCATGCGATCCTCGCAGCGTCGGCGCC-3'
HD2(Reverse primer)	5'-CGCTCGAGTCGGCTTCATCTCCCGGCCCTTAC-3'

### Sequence analysis of *Et*CHP559

The full-length cDNA sequence of the putative *Et*CHP559 gene was analyzed for similarity using the BLAST programs at NCBI (http://www.ncbi.nlm.nih.gov/BLAST/) and the genome sequence of *E*. *tenella* (http://www.genedb.org/Homepage/Etenella). The predicted amino acid sequence was obtained using the ORF Finder at NCBI (http://www.ncbi.nlm.nih.gov/gorf/gorf.html). The molecular mass, and theoretical isoelectric point were acquired using ProtParam tool at the ExPASy server (http://web.expasy.org/protparam/). Signal peptides, transmembrane regions and protein motifs were predicted using SignalP (http://www.cbs.dtu.dk/services/SignalP/), TMHMM (http://www.cbs.dtu.dk/services/TMHMM-2.0/), and Motifscan (http://hits.isb-sib.ch/cgi-bin/motif_scan) computational tools, respectively.

### Quantitative reverse transcriptase PCR (qRT-PCR) of *Et*CHP559 gene transcripts

To validate the differential expression of *Et*CHP559 in the different developmental stages, Total RNA was isolated by the TRIzol reagent (Invitrogen) from the four stages of *E*. *tenella* (UO, SO, Spz and Mrz). DNA contamination was removed by DNase I(Invitrogen)treatment. The quality and quantity of total RNA were assessed as describedin above. The cDNA was generated by SuperScriptII reverse transcriptase (Invitrogen) using random primers. Quantitative RT-PCR was performed on a Realplex 4 (Eppendorf, Hamburg, Germany) using the SYBR1 Green Idye method. Four housekeeping genes of *E*. *tenella*, 18S rRNA, β-actin, GAPDH and β-tubulin, were used as reference genes for the purposes of normalization. Primers for *Et*CHP559 (RTS and RTA) and the β-tubulin gene (β-tubulinS and β-tubulinA), were designed manually by the Beacon Designer program (www.premierbiosoft.com). The primers for the 18SrRNA gene (18S and 18A), the β-actin gene (β-actinS and β-actinA) and the GAPDH gene (GAPDHS and GAPDHA) were designed as described previously [12,13]. Each reaction was carried out in triplicate, and the experiment was performed twice. The primer sequences used for the amplification of specific genes by quantitative RT-PCR are shown in Table 1.

### Expression and purification of the recombinant *Et*CHP559 protein (r*Et*CHP559) and TF protein

The *Et*CHP559 open reading frame (ORF) was amplified with HD1 and HD2 primers, with *Bam*HI(GCGGATCC) and *Xho*I (CGCTCGAG) restriction sites at the 5' and 3' ends of the fragment, respectively. The products were digested with *Bam*HI and *Xho*I, and purified with a gel extraction kit (Tiangen, Beijing, China). The purified fragments were ligated overnight into the expression vector pCold-TF (Takara), digested by the same restriction enzymes at 4°C, and transformed into competent *Escherichia coli* BL21(DE3) (Tiangen) for protein expression. The recombinant protein was harvested after induction with 1mM isopropylthio-α-D-galactoside (IPTG) (Sigma, St Louis, MO, USA) for 24 h at 16°C. The cell pellet was lysed by sonication and analyzed by 12% SDS-PAGE to confirm the distribution of expressed recombinant protein. r*Et*CHP559 protein was purified from lysate supernatants using His Bind Resin (Merck, Darmstadt, Germany). The purity of the protein was verified by 12% SDS-PAGE and the concentration of purified protein was determined by the BCA protein assay kit (Beyotime, Haimen, China), using bovine serum albumin (BSA) as a standard. The purified protein was stored in aliquots at −20°Cuntil further use. The TF protein was purified from the lysate supernatants of *E*. *coli* BL21 cells transformed with pCold-TF plasmid as same as the methods to r*Et*CHP559 protein.

### Anti-r*Et*CHP559 polyclonal serum and anti-sporozoite polyclonal serum production

The sporozoite protein were prepared using sonication according to the previous description [12]. r*Et*CHP559 protein or sporozoite protein was used to immunize a 2-month-old male rabbit by intraperitoneal injection of 200 μg protein emulsified in Freund’s complete adjuvant (Sigma) in a 1:1 mixture. The rabbits were boosted three times at 2-week intervals with proteins emulsified in Freund’s incomplete adjuvant (Sigma). Seven days after the final immunization, antiserum against r*Et*CHP559 and sporozoite protein was collected respectively. Sera collected before protein injection was used as negative sera. All polyclonal antibodies used for sporozoite invasion inhibition experiments were purified from rabbit antiserum using Protein A+G agarose (Beyotime).

### Western blot for recombinant *Et*CHP559

Recombinant *Et*CHP559 proteins were subjected to 12% SDS-PAGE and blotted onto polyvinylidene difluoride transfer membranes (Millipore, Billerica, MA, USA). After blocking with 5% (w/v) skimmed milk powder in TBS-Tween 20 [Tris–HCl 10 mM (pH7.4), NaCl 150 mM, Tween 20 0.5%], membranes were incubated with primary antibodies (sporozoite-immunized rabbit serum at 1:100dilution) or anti-His6 monoclonal antibody (at 1:1000 dilution) or naïve rabbit serum (1:100 dilution) were used as a negative control. Horseradish peroxidase (HRP)-conjugated goat anti-rabbit IgG or goat anti-mouse IgG (1:1000 dilution; Sigma) was added as the secondary antibody, and detection used diaminobenzidine (Tiangen).

### Immunofluorescence analysis of *Et*CHP559 expression

The chicken embryo fibroblast cell line, DF-1, a derivative of the East Lansing Line (ELL-0) [14], was used for the infection and immunofluorescence experiments. DF-1 cells (3 × 10^5^ cell per well) were seeded in six-well plates (Corning, NY, USA) with precoated sterile coverslips and cultured in complete medium (Dulbecco’s Modified Eagle’s Medium (DMEM) containing 10% fetal bovine serum, 100 U/mL penicillin/streptomycin, 2 mM L-glutamine) at 37°C and 5% CO_2_ for 24 h. Freshly purified sporozoites were incubated in phosphate-buffered saline (PBS) or complete medium (CM) for 2 h at 41°C, and air dried on a glass slide before fixation [12,15]. Sporozoites previously incubated in complete medium were used to infect DF-1 cells. At various times post-inoculation, the DF-1 cells were collected, and washed. Sporozoites and cells were fixed in 2% paraformaldehyde in PBS for 15 min, and permeabilized with 1% Triton X-100 in PBS for 15 min, then blocked in PBS containing 2% (w/v) BSA overnight at 4°C. A 1:100 dilution of anti-r*Et*CHP559 polyclonal antibody was added and incubated for 1 h at 37°C. A 1:400 dilution of a goat anti-rabbit IgG fluorescein-isothiocyanate-conjugated antibody (Sigma) was added, and the slides were incubated for 1 h at 37°C. The nuclei were stained by incubation in 10 μg/mL of 4,6-diamidino-2-phenylindole (Beyotime) for 20 min. After each step described above, the slides were washed three times with PBS containing 0.05% Tween 20. The slides were treated with 60 μL Fluoromount Aqueous Mounting Medium (Sigma) and observed with a florescence microscope (Olympus, Tokyo, Japan). Second-generation merozoites purified from the ceca of chickens were also incubated in PBS or CM for observation.

### Sporozoite invasion inhibition assay

The invasion inhibition assay was based on previous reports of sporozoites of *E*. *tenella* invading DF-1 cells [12]. DF-1 cells (2 × 10^5^ cells per well) were seeded in 24-well plates and cultured in DMEM with 10% fetal bovine serum at 37°C and 5% CO_2_ for 24 h. Freshly purified sporozoites were counted and labeled with carboxyfluorescein diacetate succinimidyl ester (Beyotime), and incubated at 37°C with 25, 50, 100, 200, 300 or 400 μg/mL purified IgG against r*Et*CHP559 for 2 h, respectively. The same quantity of IgG from naïve rabbit serum was used as a negative control and the equivalent volume of PBS as a normal control. Sporozoites (10^5^/well) were used to infect 10^5^ DF-1 cells in 24-well plates (Corning) and cultured at 41°C and 5% CO_2_ for 12 h. Cells were collected, trypsinized, washed, and analyzed by Cytomics FC500 flow cytometry (Beckman Coulter, Indianapolis, IN, USA). Controls were uninfected DF-1 cells. Infected cells, uninfected cells, and free sporozoites were gated using CXP software for counting infected (labeled sporozoites) and uninfected (fluorescence-free) cells. All assays were performed in triplicate. Deduced percentages of infected cells in the presence or absence of inhibitory antibody were used to calculate inhibition rates, as described previously [14,16].

### Immunization and challenge infection

As shown in Table 2, a total of 80 one-week-old chickens were randomly divided into four groups of 20 chickens with equal mean body weights. The experimental group was immunized with 100 μg *Et*CHP559 recombinant protein emulsified in Montanide ISA 71 adjuvant (Seppic, Puteaux, France) as a 3:7 mixture per chicken, the TF protein control group was given 100 μg of TF protein emulsified in Montanide ISA 71 adjuvant as a 3:7 mixture. The chickens of the challenged control and unchallenged control groups were injected with PBS. A booster immunization was carried out 8days later with the same dose as the primary immunization. Eight days after the second immunization, blood samples were collected to isolate the serum, which was used for the detection of antibody and cytokines. Then, all the chickens except the unchallenged control group, were challenged orally with 1×10^4^ sporulated oocysts of *E*. *tenella*. While chickens of unchallenged control were given equal volume PBS orally. The fecal samples from each group were collected separately from 5^th^ day to 8^th^ day post challenge. The 9^th^ day after the challenge, all chickens were weighed and slaughtered. The cecal content for each group was collected separately. Average body weight gain, oocyst decrease ratio, lesion score, and anti-coccidial index (ACI) were calculated as follows.

**Table 2:** Protective efficacies of r*Et*CHP559 protein against an *E*. *tenella* infections.

Groups	Average body weight gains (g)	Mean lesion scores	Oocyst shedding per bird (*10^7^)	Oocyst decrease ratio (%)	Anti-coccidial index
Unchallenged control	335.35±65.26^a^	0.00±0.00^a^	0.00±0.00^a^	100^a^	200
Challenged control	237±62.15^b^	2.8±1.15^b^	9.59±2.52^b^	0.00^b^	102.67
rEtCHP559 protein	291.45±50.73^c^	1.61±0.86^c^	3.32±1.35^c^	65.36^c^	160.81
TFprotein control	245.25±57.92^b^	2.45±1.14^b^	7.67±2.36^b^	20.00^d^	108.63

^a-d^ Values with different letters in the same column are significantly different (P<0.05) according to the ANOVA Duncan test.

### Evaluation of immune protection

Protective efficacy was evaluated by the average body weight gain, oocyst output, oocyst decrease ratio, lesion score and ACI. Body weight gain was determined by subtracting the body weight at the time of challenge from the body weight at the end of the experiments. The cecal lesion scores were determined on a graded scale from 0 (normal) to 4 (severe) described by Johnson and Reid [17]. Oocysts per gram of fecal sample and cecal content were counted using McMaster’s technique. Oocyst decrease ratio was calculated as follows: (the number of oocysts from the positive control chickens − immunized chickens)/positive control chickens × 100% [18,19]. ACI is a synthetic criteria for determination of the anticoccidial effect and calculated as follows: (relative rate of weight gain + survival rate) − (lesion value + oocyst value) [20]. According to Merck Sharp & Dohme [21], ACI ≥180 is considered high performance, ACI 160–179 is considered effective, and ACI <160 is considered ineffective.

### Determination of serum antibody level by enzyme-linked immunosorbent assay (ELISA)

The serum IgG antibody responses were measured by ELISA, as described previously [22]. Briefly, the 96-well microtiter plate (Corning) was coated with 100 μL/well (10 μg/mL) r*Et*CHP559 antigen in 0.05 M carbonate buffer (pH 9.6) and incubated overnight at 4°C. After three washes with PBS containing 0.05% Tween-20 (PBST), the plate was blocked with blocking buffer (PBS containing 0.05% BSA) for 2 h at 37°C, and then the wells were washed with PBST, 100 μL/well serum samples diluted 1:100 in PBST were added, and incubated for 2 h at 37°C. Then, the plate was washed, and 50 μL/well HRP-donkey-anti-chicken IgG antibody (Sigma) diluted 1:1000 in dilution buffer was added and incubated for 2 h at 37°C. After washing, 3,3',5,5'-tetramethylbenzidine (TMB)-ELISA solution was used as a substrate. The OD was measured at 450 nm by a microplate spectrophotometer. All serum samples were included on one plate and tested by ELISA at the same time under the same conditions.

### Determination of cytokine concentrations

In order to evaluate the protection of immunized chickens, some serum cytokines were measured by ELISA with the Chick Cytokine ELISA Quantitation Kits (catalog numbers: CSB-E13114C, CSB-E14317C, CSB-E08550Ch, CSB-E12835C, and CSB-E04607Ch for sCD4, sCD8, IFN-γ, IL-10, and IL-17 respectively; CUSABIO, Wuhan, China), according to the manual instructions. These cytokines included soluble cluster of differentiation 4 (sCD4), soluble cluster of differentiation 8 (sCD8), interferon-γ (IFN-γ), interleukin-10 (IL-10), and interleukin-17 (IL-17). Every cytokine was tested two times.

### Statistical analysis

Statistical analysis was performed using SPSS for Windows version 22 (SPSS, Chicago, IL, USA). All data, including real-time quantitative PCR (qPCR), invasion inhibition assay results, body weight gain, lesion score, fecal oocyst output and oocyst decrease ratio, were analyzed. Differences among groups were tested by one-way analysis of variance (ANOVA) Duncan test. P<0.05 was considered significant and P<0.01 highly significant.

## Results

### Cloning and sequence analysis of *Et*CHP559 full-length cDNA

**Fig 1:**
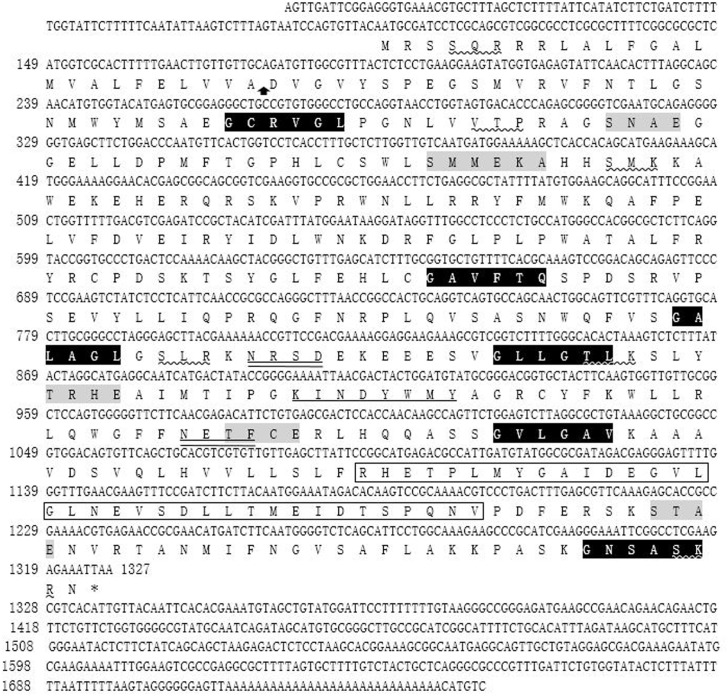
Nucleotide and predicted amino acid sequences of *Et*CHP559 gene. The stop codon is indicated with an asterisk. Putative N-glycosylation sites are double underlined. Putative protein kinase C phosphorylation sites are underlined by a wavy line. N-myristoylation sites are shaded black with white lettering. Casein kinaseII phosphorylation sites are shaded gray. Tyrosine kinase phosphorylation sites are underlined by a straight line. The vertical arrow indicates the cleavage site of the signal peptide. The ankyrin repeat is indicated by the black box.

The 1746 bp full-length cDNA was obtained by overlapping the 5'-RACE fragments with the original 701 bp EST sequence. The full-length cDNA contained a 5'-untranslated region (UTR) of 103 bp, an ORF of 1224 bp and a 3'-UTR of 419 bp (Fig 1). By analysis of the sequence, the ORF was deduced to encode a polypeptide of 407 amino acids (aa) with a calculated molecular mass of 46.04 kDa and a theoretical isoelectric point of 9.12. SignalP program analysis revealed that the Nterminus of *Et*CHP559 contained a signal peptide of 25 aa, with a hypothetical cleavage site located between alanine and aspartic acid. The *Et*CHP559 composed a cytoplasmic domain (1–8 aa), a transmembrane domain (9–31 aa) and an ectodomain (32–407 aa), as predicted using TMHMM2.0 (Fig 1). Searches in the Motif Database and Conserved Domain Database revealed the presence of two N-glycosylation sites, five casein kinase II phosphorylation sites, six N-myristoylation sites, six protein kinase C phosphorylation sites, one ankyrin repeat and one tyrosine kinase phosphorylation site, and no conserved domains were found (Fig 1).

A BLAST search of the *E*. *tenella* genome database showed that the ORF sequence shared 100% sequence identity with ETH_00024035 on superconting Eth_scaff35: 146391–147790, and that it encoded a conserved hypothetical protein. The amino acid sequence had 100% homology with the *E*. *tenella* conserved hypothetical protein (CDJ41175.1) and 92% identity to *Eimeria necatrix* hypothetical protein (CDJ65043.1) in NCBI. These demonstrated that CHP559 is a conserved protein. So, this gene was designated *Et*CHP559 (GenBank Accession No. KT318394). It also had 32% identity with *Hammondia hammondi* dense granule protein 12 (XP008888355.1) and *Toxoplasma gondii* dense granule protein 12 (ESS30190.1).

### *Et*CHP559 transcripts at different *E*. *tenella* developmental stages

**Fig 2:**
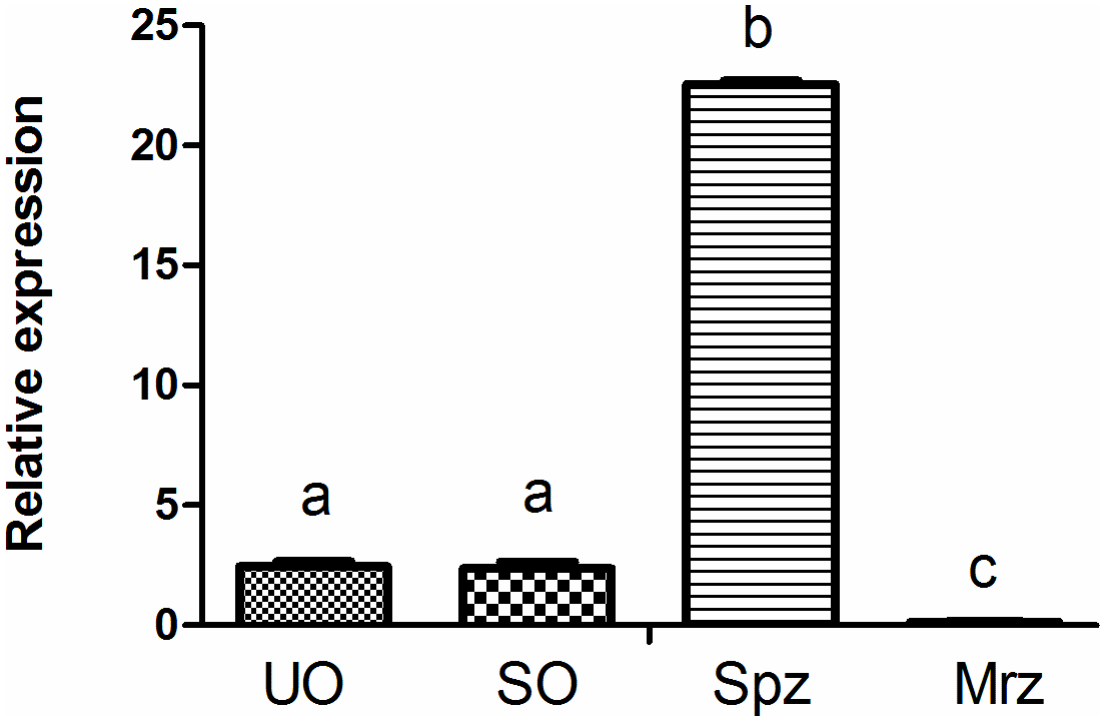
Quantitative real-time RT-PCR of EtCHP559 expression in developmental stages of *E*. *tenella*. UO, unsporulated oocysts; SO, sporulated oocysts; Spz, sporozoites; Mrz, merozoites. Bars not sharing the same letters were significantly different (P<0.05)

Real-time RT-PCR was conducted to analyze UO, SO, Spz and Mrz of *E*. *tenella* for the presence of *Et*CHP559 mRNA. The level of *Et*CHP559 mRNA in the sporozoites was much higher than that in the other three stages, and the transcripts were almost undetectable in second-generation merozoites (Fig 2).

### Expression, purification and immunoblotting of recombinant *Et*CHP559

**Fig 3:**
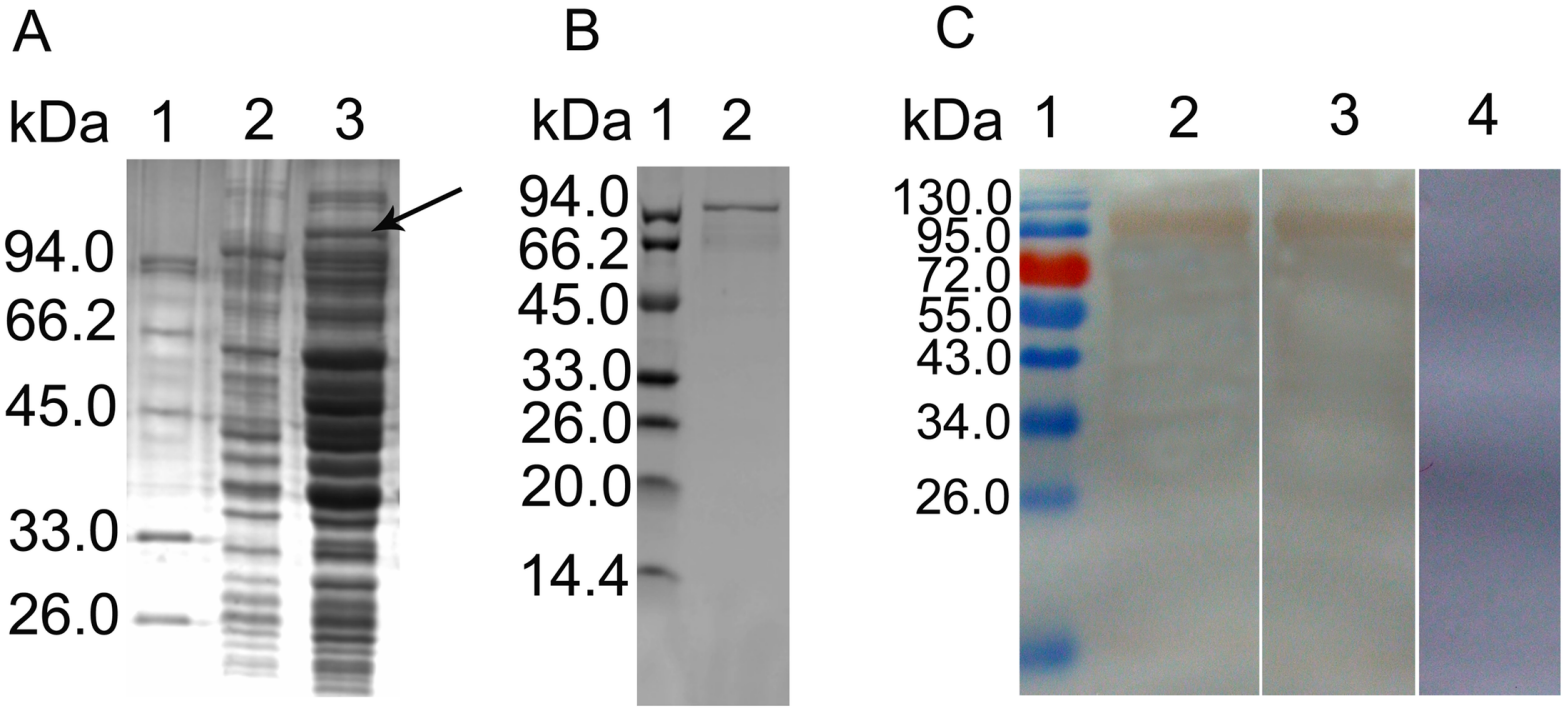
Expression and purification of rEtCHP559. (A) Proteins were analyzed by SDS-PAGE. Lane 1 protein marker, Lane 2 non-transduced *E*. *coli* lysate control, Lane 3 IPTG-induced transduced *E*. *coli* lysate. (B) Purified r*Et*CHP559 on SDS-PAGE. (C) Western blot showing r*Et*CHP559 recognition by rabbit sera against sporozoite of *E*. *tenella* or anti-His monoclonal antibody. Lane 1 protein marker, Lane 2 anti-sporozoites serum, Lane 3 anti-His monoclonal, Lane 4 naïve rabbit serum.

r*Et*CHP559 was generated as described above. SDS-PAGE showed that the recombinant protein was found in the lysate supernatants and the inclusion bodies. After purification from the supernatants by chromatography on the Ni^2+^-nitrilotriacetic acid (Ni-NTA), the protein was seen as a single band with a molecular mass of 98kDa on SDS-PAGE (Fig 3). Because 51.5 kDa of the fusion protein was from the vector and the predicted molecular mass of *Et*CHP559 protein was about 46kDa. Western blotting showed that the purified protein was recognized by rabbit serum against sporozoites or anti-His6 monoclonal antibody. Naïve rabbit sera failed to detect any protein of the expected size of r*Et*CHP559 (Fig 3). These results indicated that the r*Et*CHP559 was recognized specifically by rabbit sera against sporozoite protein and anti-His monoclonal antibody.

### Immunofluorescence of *Et*CHP559 in *E*. *tenella*-infected DF-1 cells

**Fig 4:**
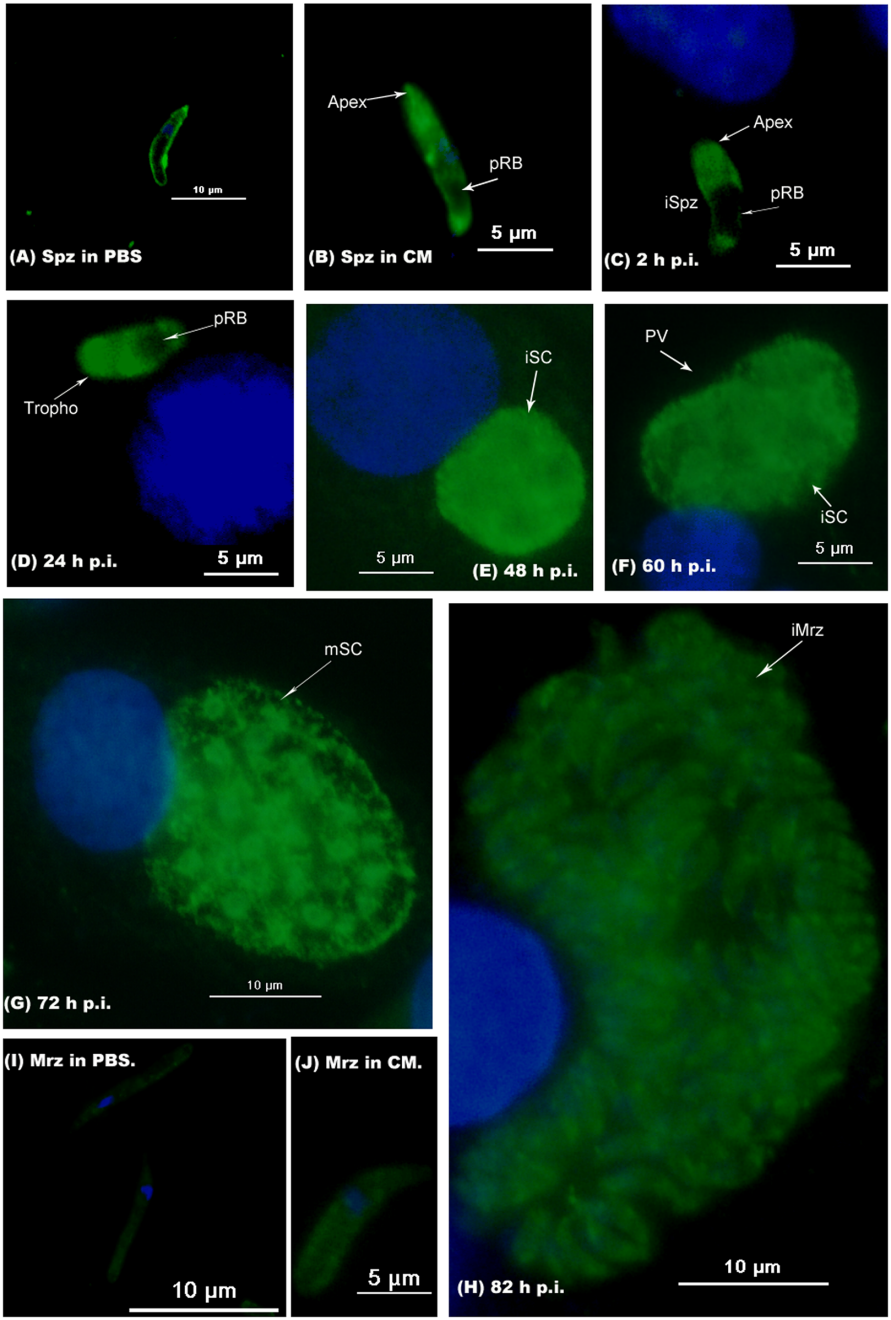
Localization of *Et*CHP559 in different stages of *E*. *tenella* by indirect immunofluorescence using r*Et*CHP559 antibody. (A) Sporozoites (Spz) in PBS; (B) Spz in complete medium. Infected DF-1 cells were collected at indicated times p.i.. (C) 2 h p.i., intracellular sporozoites (iSpz); (D) 24 h p.i., trophozoites (Tropho); (E) 48 h p.i., immature schizonts (iSC); (F) 60 h p.i., immature schizonts (iSC); (G) 72 h p.i., mature schizonts (mSC). (H) 82 h p.i., intracellular merozoites (iMrz); (I) Merozoites (Mrz) in PBS; (J) Merozoites (Mrz) in complete medium. pRB posterior refractile body, PV parasitophorous vacuole.

Using antibody against r*Et*CHP559, the localization of *Et*CHP559 in sporozoites, second-generation merozoites and during first schizogony was investigated *in vitro* by immunofluorescence. After incubation in PBS, the *Et*CHP559 protein was mainly distributed on the parasite surface of sporozoites (Fig 4A). When sporozoites were incubated in CM at 41°C for 2 h and incubated with DF-1 cells for 2 h and 24 h, *Et*CHP559 expression was increased and further distributed throughout the whole cytosol, except in the posterior refractile bodies (Fig 4B–4D). The labeled *Et*CHP559 eventually became uniformly dispersed in immature and mature schizonts (Fig 4E–4G). When the first-generation merozoites formed, staining became weaker (Fig 4H). Moreover, *Et*CHP559 was not detected in the parasitophorous vacuole (PV). When second-generation merozoites were collected from the chicken ceca and then incubated in PBS, the staining was very weak, but the staining was increased after incubated in CM at 41°C for 2 h compared to them in PBS (Fig 4I and 4J).

### Anti-r*Et*CHP559 antibodies inhibited host-cell invasion by *E*. *tenella* sporozoites

**Fig 5:**
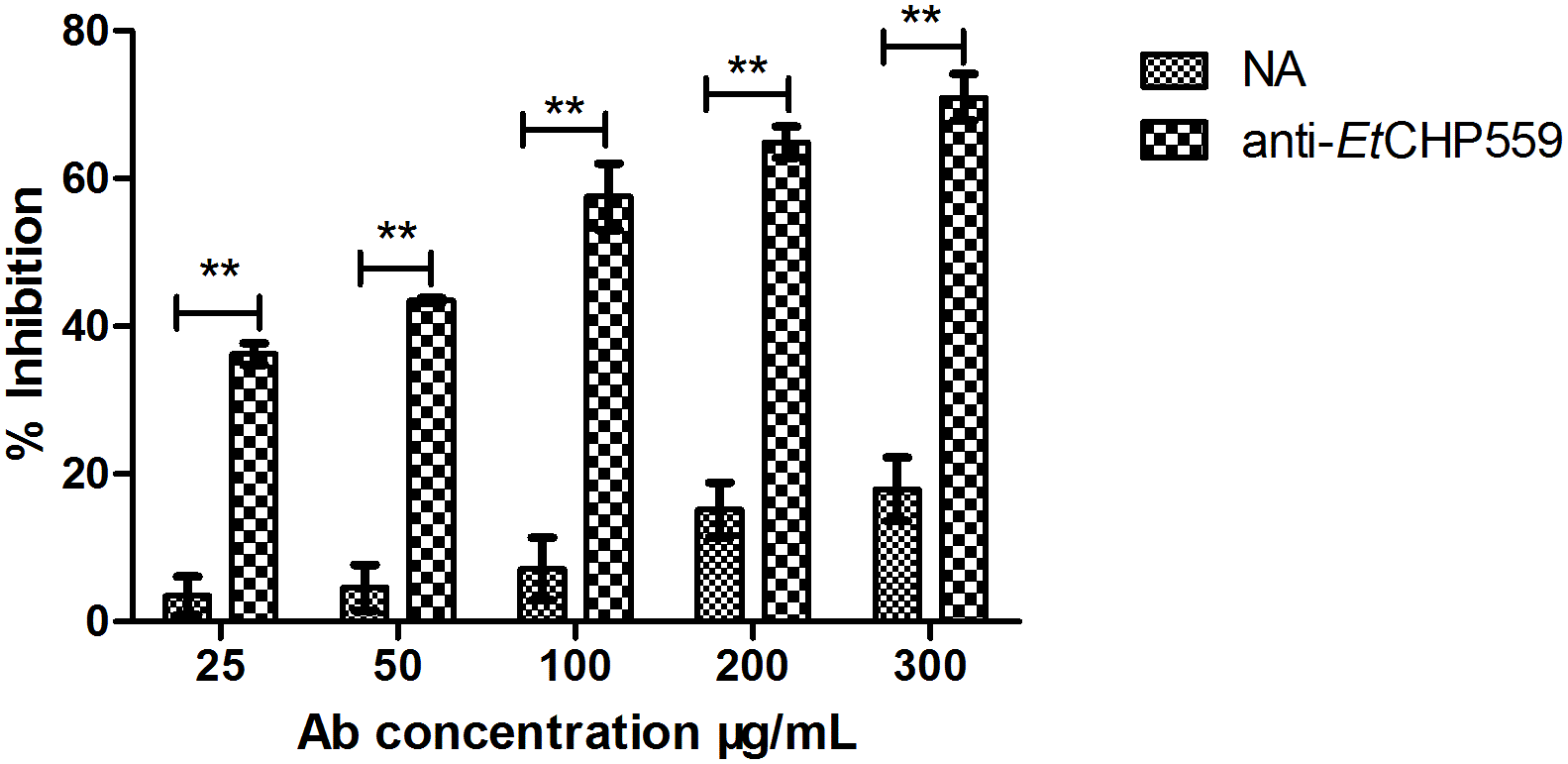
Inhibition of sporozoite invasion *in vitro* by antibody against r*Et*CHP559. Anti-rEtCHP559, rabbit antiserum against recombinant *Et*CHP559 protein; NA, naïve rabbit serum. All assays were performed in triplicate. ** P<0.01 for differences between treatment with antibody against rEtCHP559 and naïve rabbit serum at the same IgG concentration.

To test whether *Et*CHP559 protein play a role in *E*. *tenella* sporozoite invasion of DF-1 cells, we performed invasion inhibition assays of sporozoites by preincubation with r*Et*CHP559 antibody before infection. The observed inhibition effect was dose dependent (Fig 5). Pretreatment with antibody significantly affected the capacity of sporozoites to invade the DF-1 cells. Compared with the same dose of naïve rabbit sera IgG (negative control), pretreatment with 25, 50, 100, 200 or 300 μg/mL anti-*Et*CHP559 IgG highly significantly decreased invasion (P<0.01). Under the experimental conditions, an inhibition plateau of 70.9% was reached at an antibody concentration of 300 μg/mL. However, an inhibition rate of only 17.9% was found with the same dose of the naïve rabbit sera IgG, which did not have a significant effect on invasion.

### Protective effects of vaccination on *E*. *tenella* challenge

The immunization efficacies of r*Et*CHP559 are presented in Table 2. Body weight gains of the challenged control group and TF protein control group were significantly reduced compared with the unchallenged control group (P<0.05). While the body weight gains of chickens immunized with r*Et*CHP559 protein were significantly increased compared to chickens of the challenged control group and TF protein control group (P<0.05). The oocyst counts and cecal lesions of r*Et*CHP559-immunized chickens were significantly reduced compared with the challenged control group and TF protein control group (P<0.05). No chicken died from challenge infection in any groups. Chickens immunized with r*Et*CHP559 protein had an ACI >160.

### IgG titers and concentrations of cytokines, sCD4 and sCD8 in sera of immunized chickens

**Fig 6:**
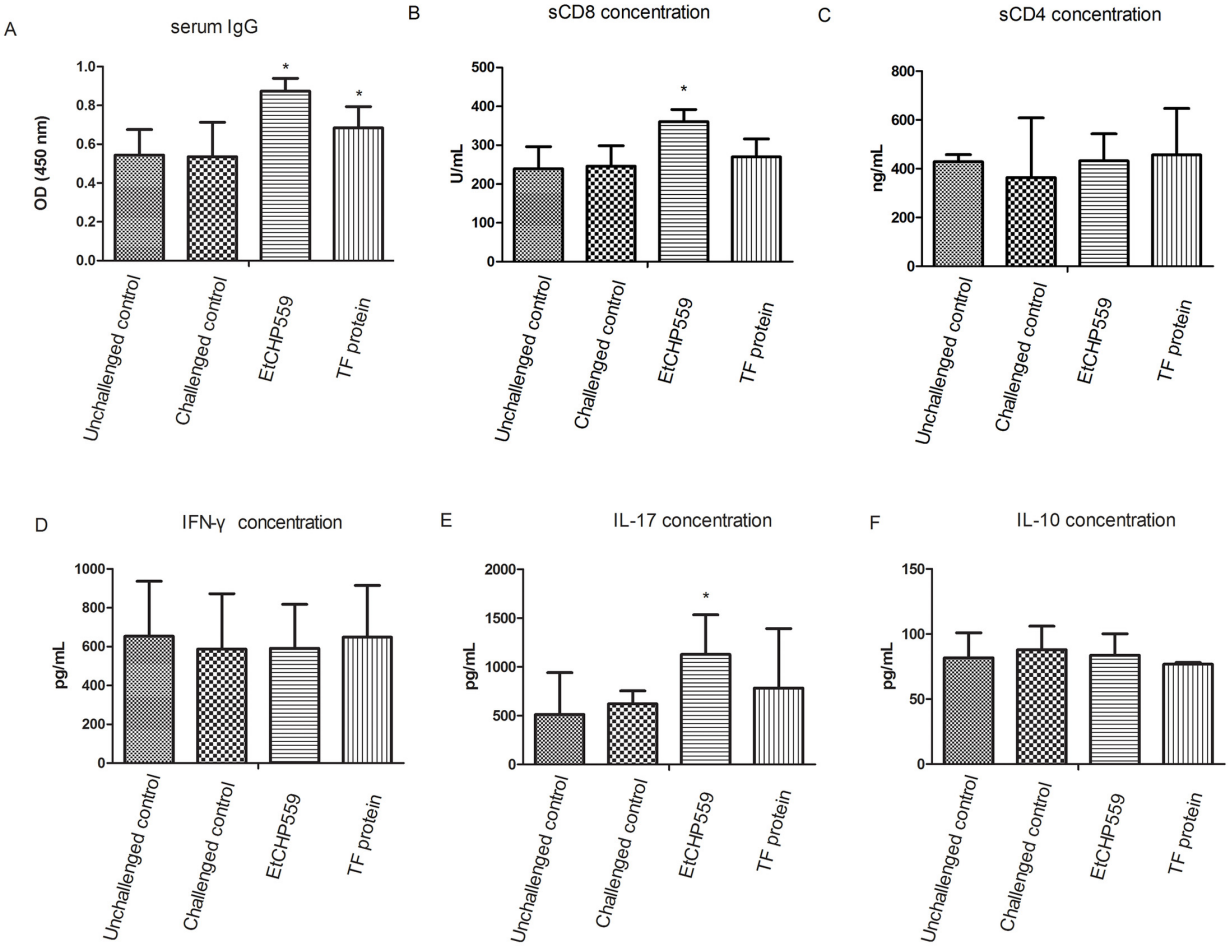
Serum r*Et*CHP559 IgG, the concentrations of sCD4, sCD8 and cytokine levels in chickens. Chickens were immunized with r*Et*CHP559 protein and TF protein. The IgG titers and the concentrations of sCD4, sCD8 and cytokine are expressed as mean ± SD. * represents a significant difference (P<0.05), otherwise there was no difference (P>0.05). A: IgG; B: sCD8; C: sCD4; D: IFN-γ; E: IL-17 and F: IL-10.

As depicted in Fig 6, significantly increased levels of r*Et*CHP559 IgG were present in r*Et*CHP559- and TF-protein-immunized chickens compared to the control groups (P<0.05). And the levels of sCD8 and IL-17 were significantly increased in chickens immunized with r*Et*CHP559 protein compared to the control groups (P<0.05). But no significant differences of sCD4, IL-10 and IFN-γ were observed between the immunized and control groups.

## Discussion

In the present work, a new gene of *E*. *tenella* was cloned and identified. The entire cDNA of this gene contained 1746 bp, which included a 1224 bp ORF encoding a 407 aa polypeptide with a predicted molecular mass of 46.04 kDa. The bioinformatics analysis predicted that the protein contained two N-glycosylation sites, five casein kinase II phosphorylation sites, six protein kinase C phosphorylation sites, and one tyrosine kinase phosphorylation site. These data suggest that its function might be regulated by post-translational modification. By BLASTP in NCBI, the deduced amino acid sequence had 100% identity with *E*. *tenella* conserved hypothetical protein (CDJ41175.1) and 92% identity with *Eimeria necatrix* conserved hypothetical protein (CDJ65043.1), but no high identity proteins were found in the other *Eimeria* spp. *E*. *tenella* and *E*. *necatrix* are the most pathogenic species among the *Eimeria* spp. and they can all reside in cecal mucosa [23]. Whether this protein is related to the virulence or parasitic site of the parasite needs to be further researched. We found that the sequence had 32% identity with *H*. *hammondi* dense granule protein 12 (XP008888355.1) and *T*. *gondii* dense granule protein 12 (ESS30190.1). The dense granule proteins are associated with the parasitophorous vacuole (PV) during and after invasion [24]. In the current research, immunofluorescence analysis using antibody against r*Et*CHP559 showed that *Et*CHP559 was not found in the PV. Whether this protein was one of the dense granule proteins needs to be further investigated.

The expression of the mRNA transcript was examined in four different development stages of *E*. *tenella*. Results from quantitative RT-PCR showed that *Et*CHP559 mRNA levels were highest in sporozoites, and the lowest was found in second-generation merozoites. This was consistent with the results of immunofluorescence, which showed that staining was weak during the second-generation merozoite stage. Previous studies of *E*. *tenella* found that the proteins that are expressed at high levels in sporozoites might participate in invasion. The protein levels of *Et*CDPK3, *Et*AMA1 and *Et*serpin are highest in sporozoites, and indirect immunofluorescence and invasion inhibition assays support their essential role in host invasion [12,14,25]. Both the sporozoites and merozoites of *E*. *tenella* are invasive for cells of the chicken cecum epithelium[26]. The previous proteomic comparison of *E*. *tenella* found that *Et*RON2 was found in sporozoites and merozoites but *Et*RON4 only in merozoites and *Et*RON5 only in sporozoites. All these proteins are related to invasion, and this suggests that the invasive mechanisms differ between merozoites and sporozoites[27]. It is suggested that *Et*CHP559 is an essential component in the invasive process of sporozoites.

Using the anti-r*Et*CHP559 polyclonal antibody, the localization of *Et*CHP559 in intracellular parasites was investigated. *Et*CHP559 was found on the parasite surface in free sporozoites incubated in PBS. The bioinformatics analysis predicted that *Et*CHP559 contained a transmembrane region and six N-myristoylation sites. Myristoylation plays a vital role in signal transduction and membrane targeting in plant responses to environmental stress [28]. These suggested that *Et*CHP559 was expressed as a membrane protein in this period. However, the *Et*CHP559 appeared to mainly concentrate in the apical complex of sporozoites after incubation in complete medium or invasion of DF-1 cells. *Eimeria* is well known for its invasion process via the apical complex present at the apex of invasive forms [29]. When sporozoites come into contact with a host cell surface, a signal is transduced from the surface to the apex, and these processes are involved in the host cell invasion [30]. The vary processes of *Et*CHP559 localization suggest that *Et*CHP559 plays a structural role in the signal transduction pathways and this needs to be further studied. Later, the specific staining of *Et*CHP559 becomes uniformly dispersed in immature and mature schizonts, but decreases in first-generation merozoites, and the staining is weak in the second-generation merozoites. This phenomenon might be that the protein expression of *Et*CHP559 in merozoites were little. This was consistent with the results of quantitative RT-PCR, which showed that *Et*CHP559 mRNA levels were the lowest in second-generation merozoites. These results show that *Et*CHP559 is also possibly related to the development of the schizont stage of *E*. *tenella*.

In this study, we found that the distribution of *Et*CHP559 in sporozoites was different between incubation in PBS and in CM. The previous research showed that *Et*Hsp90 had a homogenous distribution in the cytoplasm of sporozoites incubated in PBS and was concentrated at the apical end of sporozoites incubated in CM on the contrary. The authors also found that the secretion of *Et*Mic2 and *Et*Hsp90 was stimulated by FCS [15]. In our lab previous reports, we found that the different treatments with PBS or CM could affect the distribution of *Et*Serpin, *Et*PDIL, and *Et*eIF3s7 [12,31,32]. Moreover, the staining of *Et*CHP559 in the second-generation merozoites was increased after incubated in CM compared to them in PBS. These suggest that the CM could influence the expression and distribution of some proteins.

Previous studies have shown that apical membrane antigen (AMA)1 of *E*. *tenella* is located at the apical end of sporozoites after invasion of DF-1 cells. An invasion inhibition assay has demonstrated that antibody against r*Et*AMA1 has a remarkable inhibitory effect on parasite invasion [14]. In the present study, invasion inhibition assays revealed that rabbit antiserum against r*Et*CHP559 also blocked invasion of host cells by >70%. Combined with the results of quantitative RT-PCR and immunofluorescence localization analysis, these data supported a more direct role for *Et*CHP559 in host invasion. In our previous work, *Et*CHP559 was obtained by yeast two-hybrid screening and was assumed to be a potentially interacting protein with *Et*AMA1 (data unpublished). Although the localization of *Et*CHP559 and *Et*AMA1 in intracellular parasites overlapped, the Co-Immunoprecipitation and bimolecular fluorescence complementation assays suggested that there was no interaction of *Et*CHP559 and *Et*AMA1 (data not shown). The results show that the yeast two-hybrid assay generates false-positive interaction: protein–protein interaction that occurs in yeast cells, but not in the other cells, because the protein modification in yeast cells may not be the same as in other cells [33].

Animal experiments showed that r*Et*CHP559 provided protection against an immune challenge by significantly improving the body weight gains, reducing oocyst shedding and alleviating cecal lesions compared to controls. The ACI is commonly used to evaluate the anticoccidial effect [34]. Previous studies of *Eimeria* spp. proteins have shown that ACI can accurately reflect the protective effect [35–38]. In the present study, r*Et*CHP559 resulted in an ACI >160 in the animal protective experiment, showing partial protection against *E*. *tenella* challenge. Chickens immunized with TF protein did not show any significant protection, indicating that the immune protection against the challenge was produced by r*Et*CHP559.

The CD4^+^ and CD8^+^ lymphocytes can release soluble sCD4 and sCD8 antigens, respectively [39]. The concentrations of sCD4 and sCD8 in serum are consistent with the count of CD4^+^ and CD8^+^ lymphocytes [40]. And sCD8 is a sensitive and specific parameter of cytotoxic and suppressor T cell activation [41]. In a previous study, a gradual increase in sCD8 levels was reported in patients with malaria, which was related to malaria-associated immunosuppression [42]. In our study, the concentration of sCD8 increased significantly in chickens that were immunized by r*Et*CHP559. This suggested that r*Et*CHP559 could stimulate the recruitment of the T-cell subpopulation. IL-17 is produced by the T helper 17 subset of CD4^+^ T cells and plays a critical role in host defense against pathogens [43]. A previous study showed that IL-17 was involved in the initiation and migratory response of epithelial cells harboring *E*.*tenella* second-generation schizonts during intracellular development and contributed to the maturation of *E*. *tenella* schizonts [44]. In the present study, the concentration of IL-17 was significantly increased during immunization with r*Et*CHP559, and the staining of *Et*CHP559 became uniformly dispersed in the mature schizonts stages in the immunofluorescence assay. These data suggested that *Et*CHP559 played a role in schizont maturation. IL-10 has been shown to be crucial for control of *Eimeria* infections and plays a crucial role in preventing the development of strong IFN-γ-driven responses [45]. However, there was no significantly higher concentrations of IL-10 observed in groups immunized with r*Et*CHP559. IFN-γ is reported to be associated with protective immune responses to avian coccidiosis [46]. The CD8^+^ T lymphocytes can secret high levels of IFN-γ in response to the parasite [47], but IL-17 inhibits the expression of IFN-γ in infection and the immune response [48]. In the present study, there was no significant level of IFN-γ detected and the inconsistency of sCD8 and IL-17 needs to be further researched.

In our study, we determined the serum IgG titers of chickens immunized with r*Et*CHP559 and TF protein, and the results showed a significantly high level of IgG compared to the other chickens. In the animal experiments, the experimental group was immunized with *Et*CHP559 recombinant protein which included TF protein and the TF protein control group was immunized with TF protein, respectively. When we measured the serum IgG antibody by ELISA, the antigen used to coat 96-well microtiter plate was purified recombinant *Et*CHP559 protein which contained the TF. Because TF is a 48-kDa protein whose molecular mass is more than that of *Et*CHP559 [49]. So the TF immunization could also raise the r*Et*CHP559 IgG level. Meanwhile western blotting revealed that r*Et*CHP559 was detected in the sera of rabbits immunized with *E*. *tenella* sporozoite protein. This indicates that *Et*CHP559 can be recognized by the host immune system and enhances the humoral response.

In summary, we successfully obtained the full sequence of a novel gene *Et*CHP559 using RACE technique. We expressed and characterized this protein. Our results indicate that the protein has an important role in host cell invasion and induces partial protection against an *E*. *tenella* challenge. However, the exact roles of *Et*CHP559 in cell invasion need to be further researched.

## References

[1] Chapman HD, Barta JR, Blake D, Gruber A, Jenkins M, Smith NC, et al (2013) A selective review of advances in coccidiosis research. Adv Parasitol 83: 93–171. 10.1016/B978-0-12-407705-8.00002-1 23876872

[2] Shirley MW, Smith AL, Tomley FM (2005) The biology of avian Eimeria with an emphasis on their control by vaccination. Adv Parasitol 60: 285–330. 16230106 10.1016/S0065-308X(05)60005-X

[3] Shirley MW, Smith AL, Blake DP (2007) Challenges in the successful control of the avian coccidia. Vaccine 25: 5540–5547. 17224208 10.1016/j.vaccine.2006.12.030

[4] Blackman MJ, Bannister LH (2001) Apical organelles of Apicomplexa: biology and isolation by subcellular fractionation. Mol Biochem Parasitol 117: 11–25. 11551628 10.1016/s0166-6851(01)00328-0

[5] Blake DP (2015) Eimeria genomics: Where are we now and where are we going? Vet Parasitol 212: 68–74. 10.1016/j.vetpar.2015.05.007 25986325

[6] Reid AJ, Blake DP, Ansari HR, Billington K, Browne HP, Bryant J, et al (2014) Genomic analysis of the causative agents of coccidiosis in domestic chickens. Genome Research 24: 1676–1685. 10.1101/gr.168955.113 25015382 PMC4199364

[7] Huang B, Zhao QP, Wu XZ, Shi TW, Chen ZG (1993) Study on the identification and pathogenicity of the pure species of *Eimeria tenella*. Shanghai J Anim Husb Vet Med (in Chinese): 18–20.

[8] Tomley F (1997) Techniques for isolation and characterization of apical organelles from *Eimeria tenella* sporozoites. Methods 13: 171–176. 9405200 10.1006/meth.1997.0509

[9] Shirley MW (1995) Eimeria species and strains of chickens Eckert J., Braun R., Shirley M.W., Coudert P. (Eds.), Biotechnology—Guidelines on Techniques in Coccidiosis Research, The European Commission DGXII, Luxembourg City, Luxembourg (1995), p. 24

[10] Han HY, Lin JJ, Zhao QP, Dong H, Jiang LL, Xu MQ, et al (2010) Identification of differentially expressed genes in early stages of *Eimeria tenella* by suppression subtractive hybridization and cDNA microarray. Journal of Parasitology 96: 95–102. 10.1645/GE-2221.1 19747019

[11] Zhou BH, Wang HW, Wang XY, Zhang LF, Zhang KY, Xue FQ (2010) *Eimeria tenella*: effects of diclazuril treatment on microneme genes expression in second-generation merozoites and pathological changes of caeca in parasitized chickens. Exp Parasitol 125: 264–270. 10.1016/j.exppara.2010.01.022 20138868

[12] Jiang L, Lin J, Han H, Zhao Q, Dong H, Zhu S, et al (2012) Identification and partial characterization of a serine protease inhibitor (serpin) of *Eimeria tenella*. Parasitol Res 110: 865–874. 10.1007/s00436-011-2568-0 21842392

[13] Wang C, Han C, Li T, Yang D, Shen X, Fan Y, et al (2013) Nuclear translocation and accumulation of glyceraldehyde-3-phosphate dehydrogenase involved in diclazuril-induced apoptosis in *Eimeria tenella* (*E*. *tenella*). Vet Res 44: 29 10.1186/1297-9716-44-29 23651214 PMC3655105

[14] Jiang L, Lin J, Han H, Dong H, Zhao Q, Zhu S, et al (2012) Identification and characterization of *Eimeria tenella* apical membrane antigen-1 (AMA1). PLOS One 7: e41115 10.1371/journal.pone.0041115 22829917 PMC3400601

[15] Peroval M, Pery P, Labbe M (2006) The heat shock protein 90 of *Eimeria tenella* is essential for invasion of host cell and schizont growth. Int J Parasitol 36: 1205–1215. 16753167 10.1016/j.ijpara.2006.04.006

[16] Jahn D, Matros A, Bakulina AY, Tiedemann J, Schubert U, Giersberg M, et al (2009) Model structure of the immunodominant surface antigen of *Eimeria tenella* identified as a target for sporozoite-neutralizing monoclonal antibody. Parasitol Res 105: 655–668. 10.1007/s00436-009-1437-6 19387686

[17] Johnson J, Reid WM (1970) Anticoccidial drugs: lesion scoring techniques in battery and floor-pen experiments with chickens. Exp Parasitol 28: 30–36. 5459870 10.1016/0014-4894(70)90063-9

[18] Rose ME, Mockett AP (1983) Antibodies to coccidia: detection by the enzyme-linked immunosorbent assay (ELISA). Parasite Immunol 5: 479–489. 6355983 10.1111/j.1365-3024.1983.tb00762.x

[19] Talebi A, Mulcahy G (2005) Partial protection against *Eimeria acervulina* and *Eimeria tenella* induced by synthetic peptide vaccine. Exp Parasitol 110: 342–348. 15878770 10.1016/j.exppara.2005.03.026

[20] McManus EC, Campbell WC, Cuckler AC (1968) Development of resistance to quinoline coccidiostats under field and laboratory conditions. J Parasitol 54: 1190–1193. 5757693

[21] Ltd MSDC (1998) The control of coccidiosis: MSD Technical Booklet. 2–4 p.

[22] Lillehoj HS, Ding X, Quiroz MA, Bevensee E, Lillehoj EP (2005) Resistance to intestinal coccidiosis following DNA immunization with the cloned 3-1E Eimeria gene plus IL-2, IL-15, and IFN-gamma. Avian Dis 49: 112–117. 15839423 10.1637/7249-073004R

[23] Sharma S, Azmi S, Iqbal A, Nasirudullah N, Mushtaq I (2015) Pathomorphological alterations associated with chicken coccidiosis in Jammu division of India. J Parasit Dis 39: 147–151. 10.1007/s12639-013-0302-9 26063989 PMC4456540

[24] Nam HW (2009) GRA proteins of *Toxoplasma gondii*: maintenance of host-parasite interactions across the parasitophorous vacuolar membrane. Korean J Parasitol 47 Suppl: S29–S37. 10.3347/kjp.2009.47.S.S29 19885333 PMC2769213

[25] Han HY, Zhu SH, Jiang LL, Li Y, Dong H, Zhao QP, et al (2013) Molecular characterization and analysis of a novel calcium-dependent protein kinase from *Eimeria tenella*. Parasitology 140: 746–755. 10.1017/S0031182012002107 23369433

[26] Tabares E, Ferguson D, Clark J, Soon PE, Wan KL, Tomley F (2004) *Eimeria tenella* sporozoites and merozoites differentially express glycosylphosphatidylinositol-anchored variant surface proteins. Mol Biochem Parasitol 135: 123–132. 15287593 10.1016/j.molbiopara.2004.01.013

[27] Lal K, Bromley E, Oakes R, Prieto JH, Sanderson SJ, Kurian D, et al (2009) Proteomic comparison of four *Eimeria tenella* life-cycle stages: unsporulated oocyst, sporulated oocyst, sporozoite and second-generation merozoite. Proteomics 9: 4566–4576. 10.1002/pmic.200900305 19795439 PMC2947549

[28] Podell S, Gribskov M (2004) Predicting N-terminal myristoylation sites in plant proteins. Bmc Genomics 5: 37 15202951 10.1186/1471-2164-5-37 PMC449705

[29] Del CE, Gallego M, Sanchez-Acedo C, Lillehoj HS (2007) Expression of flotillin-1 on *Eimeria tenella* sporozoites and its role in host cell invasion. J Parasitol 93: 328–332. 17539416 10.1645/GE-992R.1

[30] Dubremetz JF, Garcia-Reguet N, Conseil V, Fourmaux MN (1998) Apical organelles and host-cell invasion by Apicomplexa. Int J Parasitol 28: 1007–1013. 9724870 10.1016/s0020-7519(98)00076-9

[31] Han H, Dong H, Zhu S, Zhao Q, Jiang L, Wang Y, et al (2014) Molecular Characterization and Analysis of a Novel Protein Disulfide Isomerase-Like Protein of *Eimeria tenella*. PLOS ONE 9: e99914 10.1371/journal.pone.0099914 24932912 PMC4059736

[32] Han H, Kong C, Dong H, Zhu S, Zhao Q, Zhai Q, et al (2015) Molecular characterization and functional analysis of subunit 7 of eukaryotic initiation factor 3 from *Eimeria tenella*. Exp Parasitol 154: 118–126. 10.1016/j.exppara.2015.04.002 25888243

[33] Vidalain PO, Boxem M, Ge H, Li S, Vidal M (2004) Increasing specificity in high-throughput yeast two-hybrid experiments. Methods 32: 363–370. 15003598 10.1016/j.ymeth.2003.10.001

[34] Li GQ, Kanu S, Xiang FY, Xiao SM, Zhang L, Chen HW, et al (2004) Isolation and selection of ionophore-tolerant Eimeria precocious lines: *E*. *tenella*, *E*. *maxima* and *E*. *acervulina*. Vet Parasitol 119: 261–276. 15154593 10.1016/j.vetpar.2003.12.009

[35] Chen P, Lv J, Zhang J, Sun H, Chen Z, Li H, et al (2015) Evaluation of immune protective efficacies of *Eimeria tenellaEt*Mic1 polypeptides with different domain recombination displayed on yeast surface. Exp Parasitol 155: 1–7. 10.1016/j.exppara.2015.04.020 25956946

[36] Zhang J, Chen P, Sun H, Liu Q, Wang L, Wang T, et al (2014) Pichia pastoris expressed *Et*Mic2 protein as a potential vaccine against chicken coccidiosis. Vet Parasitol 205: 62–69. 10.1016/j.vetpar.2014.06.029 25047705

[37] Huang J, Zhang Z, Li M, Song X, Yan R, Xu L, et al (2015) Immune protection of microneme 7 (*Em*MIC7) against *Eimeria maxima* challenge in chickens. Avian Pathol 44: 392–400. 10.1080/03079457.2015.1071780 26181095

[38] Zhang Z, Huang J, Li M, Sui Y, Wang S, Liu L, et al (2014) Identification and molecular characterization of microneme 5 of *Eimeria acervulina*. PLOS One 9: e115411 10.1371/journal.pone.0115411 25531898 PMC4274027

[39] Zajkowska J, Hermanowska-Szpakowicz T, Swierzbinska R (2001) Concentration of soluble CD4, CD8 and CD25 receptors in early localized and early disseminated Lyme borreliosis. Infection 29: 71–74. 11339478 10.1007/s15010-001-1078-x

[40] Willsie SK, Herndon BL, Miller L, Dew M (1996) Soluble versus cell-bound CD4, CD8 from bronchoalveolar lavage: correlation with pulmonary diagnoses in human immunodeficiency virus-infected individuals. J Leukoc Biol 59: 813–816. 8691065 10.1002/jlb.59.6.813

[41] Orditura M, De Vita F, Roscigno A, Auriemma A, Infusino S, Catalano G (1998) Soluble interleukin-2 receptor and soluble CD8 antigen levels in serum from patients with solid tumors. Int J Mol Med 2: 75–79. 9854147 10.3892/ijmm.2.1.75

[42] Harpaz R, Edelman R, Wasserman SS, Levine MM, Davis JR, Sztein MB (1992) Serum cytokine profiles in experimental human malaria. Relationship to protection and disease course after challenge. J Clin Invest 90: 515–523. 1644922 10.1172/JCI115889 PMC443129

[43] Iwakura Y, Ishigame H, Saijo S, Nakae S (2011) Functional specialization of interleukin-17 family members. Immunity 34: 149–162. 10.1016/j.immuni.2011.02.012 21349428

[44] Del CE, Gallego M, Lillehoj HS, Quilez J, Lillehoj EP, Ramo A, et al (2014) IL-17A regulates *Eimeria tenella* schizont maturation and migration in avian coccidiosis. Vet Res 45: 25 10.1186/1297-9716-45-25 24571471 PMC3975951

[45] Rothwell L, Young JR, Zoorob R, Whittaker CA, Hesketh P, Archer A, et al (2004) Cloning and characterization of chicken IL-10 and its role in the immune response to *Eimeria maxima*. J Immunol 173: 2675–2682. 15294985 10.4049/jimmunol.173.4.2675

[46] Lillehoj HS, Min W, Dalloul RA (2004) Recent progress on the cytokine regulation of intestinal immune responses to Eimeria. Poult Sci 83: 611–623. 15109059 10.1093/ps/83.4.611

[47] Rodriguez P, Carlier Y, Truyens C (2012) Activation of cord blood myeloid dendritic cells by *Trypanosoma cruzi* and parasite-specific antibodies, proliferation of CD8+ T cells, and production of IFN-gamma. Med Microbiol Immunol 201: 157–169. 10.1007/s00430-011-0217-y 22037700

[48] Zhang L, Liu R, Song M, Hu Y, Pan B, Cai J, et al (2013) *Eimeria tenella*: interleukin 17 contributes to host immunopathology in the gut during experimental infection. Exp Parasitol 133: 121–130. 10.1016/j.exppara.2012.11.009 23201216

[49] Saini P, Wani SI, Kumar R, Chhabra R, Chimni SS, Sareen D (2014) Trigger factor assisted folding of the recombinant epoxide hydrolases identified from C. pelagibacter and S. nassauensis. Protein Expr Purif 104C: 71–84. 10.1016/j.pep.2014.09.004 25229949

